# The Influence of Reward on Facial Mimicry: No Evidence for a Significant Effect of Oxytocin

**DOI:** 10.3389/fnbeh.2020.00088

**Published:** 2020-06-12

**Authors:** Irene Trilla, Hanna Drimalla, Malek Bajbouj, Isabel Dziobek

**Affiliations:** ^1^Berlin School of Mind and Brain, Humboldt-Universität zu Berlin, Berlin, Germany; ^2^Department of Psychology, Humboldt-Universität zu Berlin, Berlin, Germany; ^3^Digital Health Center, Hasso Plattner Institute, University of Potsdam, Potsdam, Germany; ^4^Center for Affective Neuroscience, Department of Psychiatry, Charité–Universitätsmedizin, Campus Benjamin Franklin, Berlin, Germany

**Keywords:** oxytocin, facial mimicry, reward, EMG, social modulation, null results

## Abstract

Recent findings suggest a role of oxytocin on the tendency to spontaneously mimic the emotional facial expressions of others. Oxytocin-related increases of facial mimicry, however, seem to be dependent on contextual factors. Given previous literature showing that people preferentially mimic emotional expressions of individuals associated with high (vs. low) rewards, we examined whether the reward value of the mimicked agent is one factor influencing the oxytocin effects on facial mimicry. To test this hypothesis, 60 male adults received 24 IU of either intranasal oxytocin or placebo in a double-blind, between-subject experiment. Next, the value of male neutral faces was manipulated using an associative learning task with monetary rewards. After the reward associations were learned, participants watched videos of the same faces displaying happy and angry expressions. Facial reactions to the emotional expressions were measured with electromyography. We found that participants judged as more pleasant the face identities associated with high reward values than with low reward values. However, happy expressions by low rewarding faces were more spontaneously mimicked than high rewarding faces. Contrary to our expectations, we did not find a significant direct effect of intranasal oxytocin on facial mimicry, nor on the reward-driven modulation of mimicry. Our results support the notion that mimicry is a complex process that depends on contextual factors, but failed to provide conclusive evidence of a role of oxytocin on the modulation of facial mimicry.

## Introduction

Facial mimicry is defined as the automatic imitation of emotional facial expressions of others. It is an inherent aspect of social behavior and acts as a social regulator by reinforcing social bonds and facilitating the understanding of the emotional states of others (Niedenthal, 2007; Hess and Fischer, 2013). Facial mimicry is distinguished from other affective processes that may also lead to congruent facial reactions, such as emotional contagion or affective empathy, in that mimicked facial expressions reflect the sharing of the emotional displays, rather than a response to the other’s emotional state (Hess and Blairy, 2001; Hess and Fischer, 2014).

Though initial theories understood mimicry as a stimulus-driven response, whereby the mere perception of facial expression would elicit a matching response in the observer, it is now well-established that mimicry depends on several factors related to the social context and the relationship between the interactants (Fischer and Hess, 2017). For example, pre-existing social bonds, goals to affiliate, similarity, positive mood, and a pro-social orientation have been shown to increase the tendency to mimic (see Seibt et al., 2015 for a review of social modulators of facial mimicry). These observations have motivated the notion of mimicry as a context-specific social process that occurs when there is a motivation to affiliate with the other person (Fischer and Hess, 2017) or when the interaction with the other would increase social wellbeing (Wang and Hamilton, 2012).

One of the factors shown to modulate mimicry is the reward value of the interactant. Using an implicit conditioning paradigm, Sims et al. (2012) associated different faces to losing and winning monetary rewards. Participants showed stronger facial mimicry in response to happy expressions displayed by faces previously conditioned with winning money as compared to losing money, a finding that was replicated by Korb et al. (2019). In line with these results, fMRI and EEG studies using the same reward manipulation showed a greater functional coupling between reward- and mimicry-related brain areas in response to high rewarding faces (Sims et al., 2014), as well as stronger mu suppression, considered to be an index of cortical motor simulation (Trilla Gros et al., 2015). Further support for a link between reward and facial mimicry comes from a study in which sad faces were mimicked only when participants were monetarily rewarded for accurately identifying the emotional expression (Hess et al., 2017b). In trials in which participants did not expect any reward, viewing sad faces elicited a smile instead, possibly indicative of a reaction of Schadenfreude. Altogether, these studies demonstrate that the reward value ascribed to the interactant influences the tendency to mimic their emotional expressions, arguably by impacting the implicit liking and motivation to affiliate with the person.

On the neurobiological level, oxytocin has been proposed as one endocrine factor, together with vasopressin and testosterone, influencing the modulation of mimicry (Kraaijenvanger et al., 2017). Oxytocin is a neuropeptide involved in several physiological and psychological functions that regulate both social and non-social behavior (Quintana and Guastella, 2020). Amongst other social processes, oxytocin has been shown to play a role in emotion recognition (Shahrestani et al., 2013) and empathy (Hurlemann et al., 2010). Given the role of mimicry in facilitating emotion understanding and regulating social behavior, it seems reasonable to hypothesize that some socio-cognitive effects of oxytocin could be at least partly mediated by an influence on facial mimicry. In line with this, Korb et al. (2016) tested whether intranasal administrations of oxytocin would enhance facial mimicry while making emotion judgments. Oxytocin increased facial mimicry in response to infants’ expressions of anger, but only marginally for adult targets. A small marginal increase was found for mimicry of infants’ expressions of happiness. Although these results suggest some involvement of oxytocin on facial mimicry, the effects seem to be dependent on contextual factors such as the emotion and age of the interactant.

If the role of oxytocin is to promote social adaptive behavior (Ma et al., 2016), we would expect that intranasal oxytocin enhances facial mimicry only in those contexts where there’s a motivation to affiliate with the other. Based on this, Pavarini et al. (2019) investigated whether oxytocin increased mimicry of approachable emotions (e.g., happiness, sadness) more than non-approachable emotions (e.g., fear, anger). They found that intranasal administrations of oxytocin enhanced mimicry of sadness and happiness, although the latter only in individuals who showed low positive expressivity. Concerning non-approachable emotions, no significant effects of oxytocin were found for mimicry of anger. Though these results show tentative evidence that oxytocin may selectively increase mimicry for emotions that inspire social approach, the small effect size and the complexity of these results warrant further investigation into the contextual effects of oxytocin on facial mimicry.

The current study aimed to examine the influence of oxytocin on the reward modulation of facial mimicry. Based on the idea that oxytocin promotes social adaptive behavior, and that facial mimicry preferentially occurs when we interact with rewarding others, we hypothesized that intranasal administrations of oxytocin would enhance facial mimicry as compared to placebo, but more so when viewing emotional expressions of faces associated with higher reward value.

## Materials and Methods

### Participants

Sixty healthy men (*M*_age_ = 27.40, *SD*_age_ = 6.03) were recruited for this study. Only male participants were included to avoid gender differences in oxytocin response (e.g., Lynn et al., 2014; Rilling et al., 2014). All participants were German native speakers. Exclusion criteria included a history of psychiatric or neurological disorders, heart and cardiovascular conditions, other severe medical conditions (e.g., chronic pain syndrome, chronic degenerative or inflammatory central nervous system diseases), substance use, current psychoactive medication, history of allergic or toxic reactions, smoking, participation in a pharmacological study in the last 4 months before the study, and nasal congestion or colds. Participants were asked to abstain from alcohol 24 h before the experiment, and from eating and drinking caffeine beverages for 2 h before the experiment.

All participants gave written informed consent and were financially remunerated for their participation. The study was conducted in compliance with the Code of Ethics of the World Medical Association (Declaration of Helsinki, 6th revision), and was approved by the Ethics Committee of the Department of Psychology at Humboldt-Universität zu Berlin.

### Procedure

The study followed a double-blind, placebo-controlled, between-subject design. At the start of the experimental session, participants filled out demographic information and completed the Positive and Negative Affect Schedule (PANAS; Krohne et al., 1996) to assess their current mood. Next, participants self-administered 24 IU of either oxytocin (*n* = 30) or placebo (*n* = 30) with a nasal spray. The intranasal administration was guided by the experimenter and followed the provider’s indications (Apotheke des Universitätsklinikums Heidelberg, Germany), as well as recommendations by Guastella et al. (2013). Both the experimenter and the participant were blind to the content of the nasal spray, and treatment assignment was done randomly.

Right after treatment administration, participants completed questionnaires of verbal intelligence (Mehrfachwahl-Wortschatz-Intelligenztest-B; MWT-B; Lehrl, 2005), empathy (Empathy Quotient; EQ; Baron-Cohen and Wheelwright, 2004) and autistic traits (Autism Spectrum Quotient, 33-items German version; AQ; Baron-Cohen et al., 2001; Freitag et al., 2007). Because the physiological effects of intranasal oxytocin do not begin until 30 min post-administration (Spengler et al., 2017) and questionnaire completion took a maximum of 15 min, we assumed that participants’ answers would not be affected by the treatment. During the waiting time until the next mood assessment, participants watched an affectively neutral documentary unrelated to the content of the experiment about the prehistoric monument of Stonehenge.

Thirty-five minutes after treatment administration, participants’ mood was assessed again with the PANAS. Next, participants completed a reward learning paradigm in which different neutral faces were associated with low and high reward values. After the face-reward associations were learned, participants watched videos of the same faces displaying happy and angry expressions (facial mimicry task). Electromyography (EMG) was used to track the participants’ facial expressions while watching emotion displays. Facial EMG is a widely used method for measuring facial mimicry as it allows to detect subtle face reactions that may be undetectable visually (van Boxtel, 2010). The main experimental tasks began between 39 and 50 min (*M* = 42.6, *SD* = 2.2) after treatment administration, and continued for roughly 60 min thereafter.

At the end of the session, participants completed a short questionnaire assessing blinding integrity and were debriefed about the study aims. Participants were notified about the content of the nasal spray after the data collection of the full sample was finalized.

The reward learning task was programmed in MATLAB R2015b (The MathWorks, Inc., Natick, MA, USA) using the Psychophysics Toolbox extension (Kleiner et al., 2007). OpenSesame (Mathôt et al., 2012) was used for stimulus presentation for the mimicry task. Questionnaires were implemented online using the software package SoSci Survey (Leiner, 2018).

### Reward Learning Task

**Figure 1:**
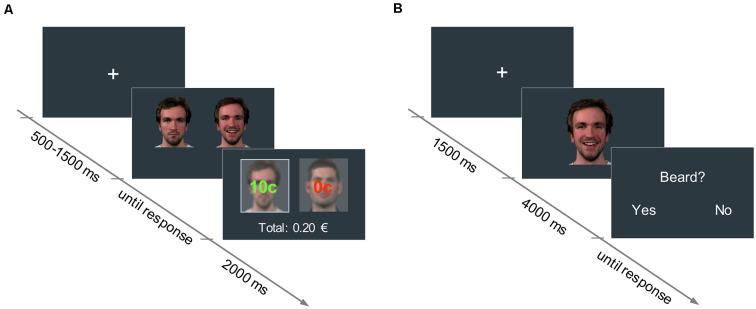
**(A)** Reward learning task: in each trial, participants had to choose between two neutral faces. One face was associated with a 60% probability of winning 10 cents (high reward condition), and the other face to a 30% probability of winning 10 cents (low reward condition). The trial outcome (i.e., “10 cents” in green, or “0 cents” in red, for the win and no-win trials, respectively) was displayed superimposed on each picture for 2,000 ms. The accumulated earnings were shown below. **(B)** Facial mimicry task: in each trial, participants watched a 4,000-ms video of a face displaying a happy or an angry expression. In 25% of the trials, participants had to answer an attention-control question concerning a physical attribute of the face they had just seen.

An associative learning paradigm based on Valentin and O’Doherty (2009) was used to pair neutral faces with low and high reward values. Pictures of four male faces with neutral expressions were selected from a validated emotion expressions database (Kliemann et al., 2013) and presented in pairs. In each trial, participants had to choose between one of the two faces displayed side by side (picture size: 400 × 400 px) by pressing either the right or left arrow key on a keyboard. Upon selection of a face, participants could either win 10 cents of a Euro (win trial) or nothing (no-win trial). For each of the two stimulus pairs, one face was associated with a 60% probability of winning (high reward condition), and the other face with a 30% probability of winning (low reward condition). The assignment of face identities to reward conditions was randomized across participants. Participants were not disclosed about the exact reward probabilities assigned to each face, but were informed that one face would lead to a higher number of win trials overall, compared to the other face in the pair. After the participant’s response, the outcome (“10 cents” or “0 cents”), as well as the accumulated earnings, were shown for 2,000 ms (see Figure 1). Participants were instructed to maximize their earnings. A fixation cross was displayed at the beginning of each trial for a variable duration between 500 and 1,500 ms.

The task consisted of a minimum of three blocks of 20 trials (10 trials per face pair, presented in random order). Participants completed up to four additional 10-trial blocks for each face pair if the proportion of high reward choices did not reach 80% by the end of each block. This learning criterion was set to make sure that the assimilation of the face-reward associations was comparable across all participants and stimuli. If participants did not reach the 80% criterium by the end of the task, the corresponding face stimuli were considered not learned and were excluded from the EMG analyses (see “EMG Analysis” section). On average, participants completed 4.20 blocks (*SD* = 1.44) per face pair. There were no significant differences in the number of blocks completed between oxytocin and placebo groups, *t*_(58)_ = 0.14, *p* = 0.89.

To check whether the reward associations changed how faces were evaluated, participants were asked to rate the pictures for pleasantness using a 7-point scale (1: very unpleasant; 7: very pleasant) before and after the reward learning task.

### Facial Mimicry Task

A passive viewing task was used to assess facial mimicry. In each trial, participants watched a 4,000 ms-video of a face displaying either a happy or an angry expression (768 × 768 px). Videos started with a neutral face that changed into the emotional expression, which peaked at around 1,500 ms. Dynamic expressions were used as they have been shown to elicit stronger facial mimicry responses than static pictures and are more ecologically valid (Sato et al., 2008). Preceding each video, a fixation cross was presented for 1,500 ms.

In total, participants watched happy and angry facial expressions of six male actors. Four of the face identities had been previously associated with either high or low reward values in the reward learning task. The remaining two identities were new to the participants (unconditioned faces) and were used as a control condition to assess the direct influence of oxytocin on facial mimicry. All videos were presented eight times (96 trials in total) in randomized order.

To assure that participants paid attention to the videos, in 25% of the trials a yes/no question about physical attributes of the actors (e.g., presence of a beard, hair color) was asked right after the clip presentation (see Figure 1). Videos of the participants during the mimicry task were recorded with a webcam to detect potential artifacts in the EMG data (e.g., if participants sneezed, moved, et cetera).

### EMG Data Acquisition

During the facial mimicry task, EMG was used to record the activity of the zygomaticus major (ZM), a muscle on the corners of the mouth that is activated when smiling, and the corrugator supercilii (CS), a muscle located in the eyebrow area that contracts when frowning. Stronger activity in the ZM as compared to CS is commonly used as an index of mimicry of happiness, while the opposite pattern (i.e., higher CS than ZM) reflects an expression congruent with anger (van Boxtel, 2010; Hess et al., 2017a). Bipolar Ag/AgCl electrodes were attached to the left side of the face over the two muscles. The ground electrode was placed on the center of the forehead, below the hairline. We followed standard EMG site preparation and electrode placement procedures (van Boxtel, 2010). Skin conductance electrode gel was used to facilitate conductance between the electrodes and the facial skin. To cover the recording of muscular activity, participants were told that facial electrodes were measuring sweat production.

EMG signals were amplified with EMG amplifiers (Becker Meditec, Karlsruhe, Germany; gain = 1,230; frequency response 19–500 Hz). The amplified signals were digitized using a USB multifunction card USB-6002 (National Instruments Inc., Ireland) connected to a laptop computer Dell Latitude 5540, running data acquisition software DasyLab 10.0 (National Instruments Ireland Resources Limited). The raw EMG signals were sampled with 500 Hz and 16-bit resolution. Within DasyLab, signals were online (RMS root mean square) integrated with a time constant of 50 ms and rectified. The multifunction card USB-6002 acquired also trigger signals from the parallel port of the presentation computer. For further processing, the integrated EMG and the trigger signals were down-sampled to 20 Hz and stored as an ASCII file.

### EMG Data Reduction and Artifact Control

EMG data was pre-processed offline in Matlab R2015b (The MathWorks, Inc., Natick, MA, USA) using self-made scripts. Data were segmented from 1,500 ms before to 4,000 ms after stimulus onset. To detect trials with artifacts, the raw EMG signal and the video recordings of all participants were screened visually. Artifacts were defined as distortions in the EMG data associated with, for example, resting the chin on the hand, swinging the head, yawning, eye closing, mumbling, and displaying facial expressions in the pre-stimulus period. On average, 3.79 trials (*SD* = 3.56) were rejected per participant. There were no significant differences in the number of trials rejected by emotion and reward conditions, *F*_(2,114)_ = 0.35, *p* = 0.71. Artifact-free data were Z-standardized within muscles and within participants to account for individual and muscle differences. Due to technical issues during EMG acquisition, data in the 500 ms directly before and after stimulus onset were distorted and could not be used. The period from 1,500 ms to 4,000 ms after stimulus onset was used as the window of interest. As in Sims et al. (2012); this interval was determined based on the time when the emotion expressions peaked during the videos (i.e., at around 1,500 ms in this study), and because it included the period of maximal EMG responses (see Figures 3, 4). Change from baseline scores were calculated for each trial and muscle by subtracting the mean EMG amplitude from 500 to 1,000 ms preceding stimulus onset (baseline) from the mean EMG amplitude of the window of interest. The resulting baseline-corrected EMG scores were used as the dependent variable in the statistical analyses.

**Figure 2:**
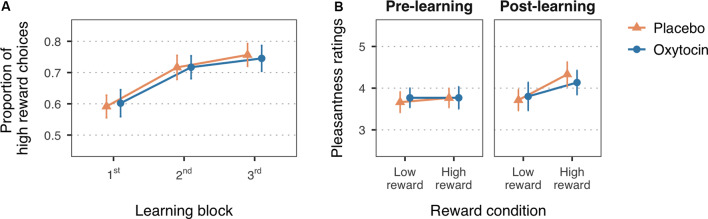
**(A)** Mean proportion of high reward choices for the first three learning blocks of the reward learning task. **(B)** Mean pleasantness ratings for the faces paired with low and high reward values, before and after the reward learning task. Error bars are within-subject 95% confidence intervals.

**Figure 3:**
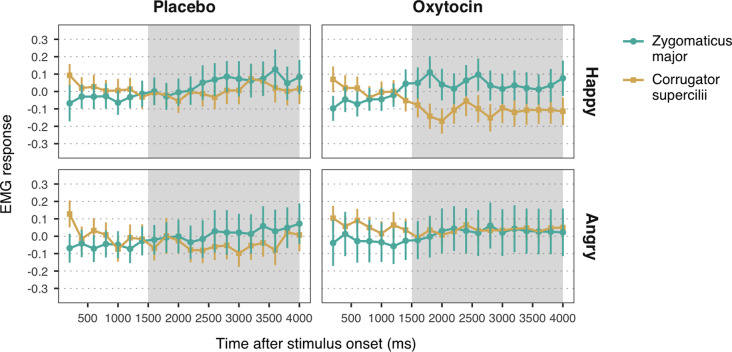
Zygomaticus major and corrugator supercilii responses to happy and angry facial expressions of unconditioned faces, for each treatment group. Plotted data corresponds to Z-standardized, baseline-corrected electromyographic (EMG) activity averaged within 200-ms time-bins. Only data from the time-window between 1,500 and 4,000 ms post-stimulus onset (shaded in gray) was used for statistical analysis. Error bars represent within-subject 95% confidence intervals.

**Figure 4:**
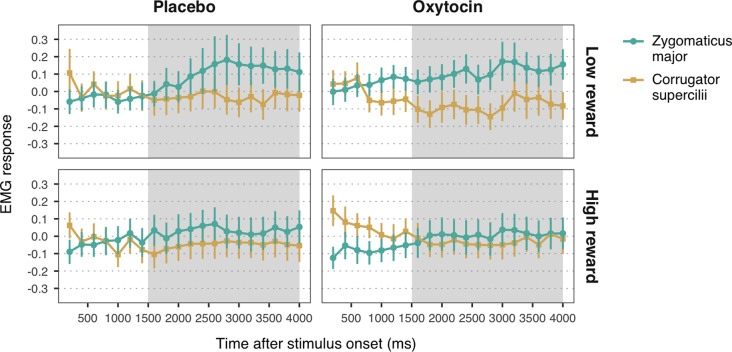
Zygomaticus major and corrugator supercilii responses to happy faces associated with low and high reward values, for each treatment group. Plotted data corresponds to Z-standardized, baseline-corrected EMG activity averaged within 200-ms time-bins. Only data from the time-window between 1,500 and 4,000 ms post-stimulus onset (shaded in gray) was used for statistical analysis. Error bars represent within-subject 95% confidence intervals.

### Statistical Analysis

Data and code necessary to reproduce the analyses reported here are available at: osf.io/n85sh. All statistical analyses were run in R (R Core Team, 2014) and R studio (RStudio Team, 2019). The main R packages used were: *afex* for ANOVA (Singmann et al., 2019); *lme4* (Bates et al., 2015), *lmerTest* (Kuznetsova et al., 2017) and *emmeans* (Lenth, 2020) for linear mixed-effects analysis; *pwr* (Champely, 2018) and *TOSTER* (Lakens, 2017) for equivalence testing; and *ggplot2* (Wickham, 2009) for figures.

#### Reward Manipulation Check

To test whether participants learned the reward associations, the proportion of high reward choices for each of the first three learning blocks were fitted in a 2 × 2 mixed ANOVA with Block (first, second, third) as within-subject factor and Treatment (placebo, oxytocin) as between-subject factor. A 2 × 2 × 2 mixed ANOVA with Reward (low reward, high reward) and Time (pre-, post-reward learning task) as within-subject factors, and Treatment (placebo, oxytocin) as between-subject factor, was used to examine whether face pleasantness ratings changed after the reward associations were learned. The Holm-Bonferroni method was applied to adjust for multiple comparisons in *post hoc* tests.

#### EMG Analysis

Due to technical problems, EMG data of two participants were not collected. Data from two additional participants were excluded from the EMG analyses due to low accuracy (less than 80%) on the attention control questions in the mimicry task. The EMG analysis sample thus included data from 29 participants in the oxytocin group and 27 participants in the placebo group.

EMG data were analyzed using linear mixed models (LMM). Separate LMMs were executed to test EMG responses to observing happy and angry expressions. Mimicry of happiness was defined as increased activation of ZM vs. CS in response to happy faces. The opposite pattern of muscular activity (i.e., stronger CS vs. ZM response) in response to angry faces would be indicative of mimicry of anger.

First, we examined the direct influence of oxytocin on facial mimicry. EMG data from trials in which participants viewed unconditioned faces (i.e., faces that did not appear in the reward learning task, and thus had not been associated to any particular reward value) were used as the dependent variable in LMMs with Muscle (ZM, CS), Treatment (placebo, oxytocin) and their interaction as fixed effects.

Second, to test the effects of reward on facial mimicry, and the modulatory role of oxytocin, we fitted into LMMs the EMG data from trials that presented low reward and high reward faces. Trials in which the face presented did not reach the learning criterion by the end of the reward learning task were excluded. The LMMs included the main effects of Muscle (ZM, CS), Reward (low reward, high reward), and Treatment (placebo, oxytocin), as well as the corresponding two- and three-way interactions, as fixed effects.

To account for non-independencies in the data, we entered by-participant and by-stimulus random intercepts in all LMMs. If a model led to singular fits, the random-intercept for stimuli was removed. Sum to zero contrasts were set for all predictors. *P*-values were computed based on Satterthwaite approximation for denominator degrees of freedom.

## Results

Overall, 37% of the participants guessed correctly the treatment that they had received, 40% made an incorrect guess, and 23% reported not knowing. The proportion of participants who made correct or incorrect guesses did not significantly differ between the placebo and oxytocin groups, $\chi_{\left(2\right)}^{2}$ = 1.49, *p* = 0.47.

With respect to mood changes, participants in both the placebo and oxytocin groups reported less negative affect after treatment administration (*M* = 12.6, *SD* = 3.97) than at baseline (*M* = 14.02, *SD* = 4.62), as shown by a significant main effect of time, *F*_(1,58)_ = 14.35, M*SE* = 4.19, *p* < 0.001, ${\hat{\eta }}_{G}^{2}$ = 0.027. No significant change in positive affect was observed, nor a significant effect of treatment for neither positive or negative affect (all *p*_s_ > 0.47). Descriptive statistics of questionnaire scores are available in the Supplementary Table S1.

### Learning of Reward Associations

In line with the expected reward learning curve, the proportion of high reward choices significantly increased with the number of blocks, *F*_(1.99,115.52)_ = 36.51, *MSE* = 0.01, *p* < 0.001, ${\hat{\eta }}_{G}^{2}$ = 0.10 (Figure 2A). Participants chose the high reward faces more often in the second (*M* = 0.72, *SD* = 0.21), *t*_(116)_ = −6.33, *p* < 0.001, Cohen’s *d* = −0.60, and third blocks (*M* = 0.75, *SD* = 0.20), *t*_(116)_ = −8.14, *p* < 0.001, Cohen’s *d* = −0.80, as compared to the first block of trials (*M* = 0.60, *SD* = 0.19). The difference between the second and third block did not reach statistical significance, *t*_(116)_ = −1.80, *p* = 0.07, Cohen’s *d* = −0.17. There was no significant main effect of treatment, *F*_(1,58)_ = 0.26, *MSE* = 0.10, *p* = 0.61, ${\hat{\eta }}_{G}^{2}$ = 0.004, nor a significant interaction with block, *F*_(1.99,115.52)_ = 0.16, *MSE* = 0.01, *p* = 0.85, ${\hat{\eta }}_{G}^{2}$ < 0.001, which is consistent with previous studies in which intranasal oxytocin did not enhance learning with non-social reinforcements as compared to placebo (Hurlemann et al., 2010; Clark-Elford et al., 2014). Descriptive statistics on the proportion of high reward choices for each block and treatment group can be found in the Supplementary Table S2.

A change in the perceived pleasantness of the faces after the reward learning task, indicated by a significant reward-by-time interaction, *F*_(1,58)_ = 8.74, *MSE* = 0.31, *p* = 0.004, ${\hat{\eta }}_{G}^{2}$ = 0.01, supports that face-reward associations were assimilated (Figure 2B). Planned simple effects confirmed that, while there were no significant differences in pleasantness ratings at baseline (low reward: *M* = 3.72, *SD* = 0.71; high reward: *M* = 3.77, *SD* = 0.89), *t*_(94.30)_ = −0.36, *p* > 0.99, Cohen’s *d* = −0.06, faces associated with high reward value were rated as more pleasant (*M* = 4.23, *SD* = 0.98) than faces associated with low reward value (*M* = 3.76, *SD* = 0.91) after the reward learning task, *t*_(94.30)_ = −3.37, *p* = 0.003, Cohen’s *d* = −0.50. Treatment group did not significantly interact with these effects, *F*_(1,58)_ = 0.41, *MSE* = 0.31, *p* = 0.53, ${\hat{\eta }}_{G}^{2}$ = 0.001, nor had an overall influence on pleasantness ratings, *F*_(1,58)_ = 1.16, *MSE* = 1.52, *p* = 0.29, ${\hat{\eta }}_{G}^{2}$ = 0.010. See Supplementary Table S3 for descriptive statistics on the pleasantness ratings for each treatment group.

### Effects of Oxytocin on Facial Mimicry

Observation of happy facial expressions elicited higher ZM (*M* = 0.05, *SE* = 0.04) compared to CS activity (*M* = −0.06, *SE* = 0.04), *b* = 0.05, *SE* = 0.02, 95% *CI* = (0.02, 0.09), *t* = 3.04, *p* = 0.002, confirming the occurrence of mimicry of happiness (Figure 3). Contrary to our expectations, the interaction between muscle and treatment was not significant, *b* = −0.03, *SE* = 0.02, 95% *CI* = (−0.06, 0.01), *t* = −1.65, *p* = 0.10. The main effect of treatment, however, was significant, *b* = 0.06, *SE* = 0.02, 95% *CI* = (0.02, 0.10), *t* = 2.71, *p* = 0.007, indicating that both ZM and CS were more activated in the oxytocin group (*M* = −0.06, *SE* = 0.04) as compared to the placebo group (*M* = 0.05, *SE* = 0.04).

With respect to responses to angry facial expressions, we did not find any significant difference between ZM and CS, *b* = 0.01, *SE* = 0.02, 95% *CI* = (−0.03, 0.06), *t* = 0.64, *p* = 0.52, which indicates that our task did not elicit mimicry of anger (Figure 3). No significant main effect of treatment, *b* = −0.003, *SE* = 0.03, 95% *CI* = (−0.05, 0.05), *t* = −0.11, *p* = 0.91, nor an interaction with muscle reactivity were found, *b* = 0.02, *SE* = 0.02, 95% *CI* = (−0.03, 0.06), *t* = 0.82, *p* = 0.41.

### Effects of Reward on Facial Mimicry and the Influence of Oxytocin

The LMM with EMG responses to high and low reward happy faces as dependent variable yielded a significant main effect of muscle, *b* = 0.06, *SE* = 0.01, 95% *CI* = (0.03, 0.08), *t* = 4.37, *p* < 0.001. As before, viewing happy facial expressions elicited stronger ZM activity (*M* = 0.07, *SE* = 0.03) than CS activity (*M* = −0.05, *SE* = 0.03), confirming mimicry of happiness. Moreover, we found a significant muscle-by-reward interaction, *b* = 0.03, *SE* = 0.01, 95% *CI* = (0.002, 0.05), *t* = 2.10, *p* = 0.036. Follow-up analyses on the estimated means showed that ZM response was higher for low reward happy faces (*M* = 0.12, *SE* = 0.03) as compared to high reward faces (*M* = 0.02, *SE* = 0.03), *t*_(2552.70)_ = 2.54, *p* = 0.03 (Figure 4). This result indicates that, contrary to our expectations, low reward happy faces elicited stronger mimicry than higher reward happy faces. No significant differences in CS activity were found between high and low reward faces, *t*_(2552.70)_ = −0.42, *p* = 0.68. Neither the predicted three-way interaction, nor any of the two-way interactions with treatment reached significance (all *p*_s_ > 0.39). These results thus provide no significant evidence for a modulation of oxytocin on the effects of reward on mimicry of happiness.

The LMM on EMG responses to angry expressions did not yield any significant main effects or interactions (all *p*s > 0.14; Figure 4). See the Supplementary Material for the descriptive statistics (Supplementary Table S4) and the complete results of the LMMs (Supplementary Tables S5, S6).

### Equivalence Testing

Because null-hypothesis significance testing can only reject the presence of an effect, we cannot conclude that intranasal oxytocin does not influence facial mimicry based on the non-significant results reported above. Using the Two One-Sided Tests (TOST) procedure of equivalence testing (Lakens, 2017), we reexamined our main null findings to test whether the effect of oxytocin on facial mimicry was statistically equivalent to the placebo effect, or whether our data was just not sensitive enough to detect the predicted group differences. We limited this analysis to EMG responses to happy faces, as our task failed to elicit mimicry of anger. Given that there is no clear theoretical boundary for oxytocin’s social-cognitive effects for setting the equivalence bounds (Quintana, 2018), we defined the smallest effect size of interest-based on the smallest effect size detectable with 80% power given our sample size (Quintana, 2018; Tabak et al., 2019). The alpha level was set to 0.05.

First, we applied the TOST procedure to test the null effect of oxytocin on EMG responses to happy expressions of unconditioned faces. Because the TOST procedure is based on *t*-tests, we reduced the original Muscle-by-Treatment interaction tested in the LMM by computing mimicry indices (i.e., difference score between CS and ZM activity in response to happy faces) for each participant. Positive scores indicate the occurrence of mimicry of happiness. The equivalence test comparing the mean mimicry index of the oxytocin vs. placebo groups was non-significant, *t*_(49.15)_ = 1.55, *p* = 0.06, given equivalence bounds of −0.76 and 0.76. This indicates that we cannot reject effects bigger than what could be reliably tested based on the statistical power of our study.

Second, we tested the oxytocin effects on the influence of reward on mimicry of happiness. To reduce the original three-way interaction in the LMM (Muscle × Reward × Treatment), we subtracted the mimicry index to high reward faces from the mimicry index to low reward faces for each participant. Positive scores indicate stronger mimicry of happiness in response to high reward vs. low reward faces. The equivalence test comparing the oxytocin and placebo groups did not reach significance, *t*_(48.98)_ = −1.64, *p* = 0.05, given equivalence bounds of −0.80 and −0.80. Note that the equivalence bounds were recalculated for this second TOST because the sample size for this analysis was smaller: data from two participants of the oxytocin group and 3 of the placebo group were not included because they did not reach the learning criterium for any of the face pairs in the reward learning task.

Based on the equivalence tests and the null-hypothesis tests combined, we can neither conclude that oxytocin has an effect on facial mimicry nor reliably reject effect sizes that could be detected with 80% power given the sample size of this study.

## Discussion

This study sought to investigate the modulatory effect of intranasal oxytocin on the link between reward and facial mimicry. We hypothesized that intranasal administration of oxytocin would increase facial mimicry, but more so in response to faces previously associated with high reward as compared to low reward value.

Our study failed to confirm our original hypotheses. First, we found an influence of reward on facial mimicry of happiness, but this effect was in the opposite direction as predicted: happy faces associated with low reward were mimicked more than happy faces associated with high reward. Second, we did not find evidence for a significant effect of oxytocin on facial mimicry, neither for a direct influence, nor on the modulation of the reward-mimicry link.

### The Influence of Reward on Facial Mimicry

Previous studies reported a higher tendency to mimic the emotional expressions of faces paired with high rewards than with low rewards (Sims et al., 2012; Hess et al., 2017b; Korb et al., 2019). Here, we manipulated the value of different neutral faces using an associative learning task with monetary rewards. No differences in the learning of face-reward associations were found between the oxytocin and placebo groups, in line with previous studies in which intranasal oxytocin did also not facilitate learning with non-social reinforcements (Hurlemann et al., 2010; Clark-Elford et al., 2014). As expected, our reward manipulation changed how participants evaluated the faces, such that those linked with higher monetary rewards were rated as more pleasant. However, the EMG results did not replicate the direction of the reward effects of previous mimicry studies, as participants showed stronger mimicry in response to happy faces previously conditioned with low reward as compared to high rewarding happy faces.

Differences in the sample characteristics and experimental design may account for this unexpected result. For example, previous studies on the reward-mimicry link had predominantly female samples, and their stimuli included both female and male targets (Sims et al., 2012; Hess et al., 2017b; Korb et al., 2019). Conversely, we only included male participants and same-gender face identities. Given that there are gender differences in how characteristics of the target person, such as gaze direction and gender, influence mimicry reactions (Schrammel et al., 2009; Hess and Bourgeois, 2010), it could be that the direction of reward effects is partly dependent on the gender of and the relationship between the expresser and the perceiver.

Our study also differed from previous ones in how reward was manipulated. In Hess et al. (2017b); the motivation to affiliate, rather than the value of the targets themselves, was manipulated by either rewarding (or not rewarding) the correct identification of their emotional expressions. In contrast, Sims et al. (2012) and the current study aimed to directly alter the value of the faces, although the paradigm and reward schemas used were different. First, Sims et al. (2012) used a classical conditioning task to implicitly pair different reward values with face identities. While this task proved to be effective in manipulating the value of the stimuli (Korb et al., 2019), it had the limitation that the participant’s learning of the associations could not be tracked, as task performance was unrelated to the associations between the face stimuli and monetary rewards. In contrast, we implemented a reward learning task in which the reward outcomes were contingent on the participant’s choices. This allowed us to use task performance as an indication of the learning of the face-reward pairings.

Second, Sims et al. (2012) compared mimicry responses to faces associated with rewards (winning money) vs. faces associated with punishments (losing money). In the current study, face stimuli were only conditioned with different probabilities of winning money. We chose relatively low reward probabilities (60% for high reward condition vs. 30% for low reward condition) to keep the learning implicit, as pilot testing suggested that at these rates participants were not aware that they had learned the associations, despite learning curves and pleasantness ratings were as expected. Note that even though reward probabilities were overall lower than those used in Sims et al. (2012); faces assigned to the high reward condition were anyway paired twice as often with a monetary outcome than faces in the low reward condition.

These variations in the reward schemas may have affected how the manipulation influenced the evaluation of the faces as compared to previous studies. Rather than associating a positive or negative valence to the face identities (e.g., targets paired with positive outcomes vs. targets paired with negative outcomes), our manipulation may have changed the level of uncertainty as to which each face would be accompanied with a rewarding outcome. According to a recent account of social cognition, the inability to precisely predict the states and actions of others during social interactions is associated with aversive feelings (FeldmanHall and Shenhav, 2019). On this account, processes of inference and affect-sharing such as emotional mimicry are thought to be activated to reduce social uncertainty. Therefore, participants may have reacted with stronger mimicry to low reward faces as a way to promote emotion understanding, regain a bond with the interactant, and ultimately reduce the higher social uncertainty that the low reward faces conveyed as compared to faces associated with higher reward probability.

### Null Effects of Intranasal Oxytocin

Contrary to our predictions, intranasal administrations of oxytocin did not influence the degree of facial mimicry of happiness, nor the modulation by reward. Two studies have previously investigated the effects of oxytocin on facial mimicry, and neither found intranasal oxytocin to consistently change the facial reactivity to happy expressions (Korb et al., 2016; Pavarini et al., 2019). Only in their exploratory analysis did Pavarini et al. (2019) detect oxytocin-related increases in mimicry reactions to happy faces in a subsample of participants who showed reduced positivity expressivity at baseline. A meta-analysis found small increases in the expression of positive emotions after intranasal oxytocin, although it did not significantly improve the recognition of happiness (Leppanen et al., 2017). Due to the sample size, our study had limited power to detect small or medium effects, a shortcoming that was further highlighted by the inconclusive results of the equivalence tests applied. While our data was not able to provide conclusive evidence on the (lack of) effects of intranasal oxytocin, cumulative null findings may indicate that, even if oxytocin would influence the degree of mimicry of happiness, this effect is probably small.

Mimicry of happiness is highly frequent and very consistent across individuals and social contexts (Bourgeois and Hess, 2008; Hess and Bourgeois, 2010), which may render an effect of oxytocin difficult to detect. Instead, oxytocin manipulations may be more prone to affect mimicry reactions to emotions that are more context- and person-dependent, such as sadness and anger. Given that our mimicry task did not elicit congruent facial responses to angry expressions, we were unable to test the effects of oxytocin and reward on mimicry for anger. However, previous studies did find oxytocin-related increases in mimicry in response to angry expressions (Korb et al., 2016), and a trend towards an increase of mimicry of sadness (Pavarini et al., 2019).

Nevertheless, current evidence of the role of oxytocin on facial mimicry remains weak. Beyond our null findings, oxytocin did not have a robust direct influence on mimicry reactions in previous studies. Rather, effects were only observed in response to certain stimuli (e.g., children’s emotional faces vs. adult faces, in Korb et al., 2016), emotional expressions (e.g., angry vs. happy in Korb et al., 2016; and sad vs. happy and angry, in Pavarini et al., 2019), and type of mimicry tasks (e.g., offset task vs. intensity task, in Korb et al., 2016). Altogether, this suggests that oxytocin may not act by directly influencing basic mechanisms underlying mimicry. Instead, it could play a role in the modulation of facial mimicry by the social context. This would fit well with current theories of oxytocin, which posit that oxytocin improves social adaptation by increasing social salience (Shamay-Tsoory and Abu-Akel, 2016) and promoting approach behavior and reducing avoidance (Harari-Dahan and Bernstein, 2014). Our results on the oxytocin modulation of the reward-mimicry link, however, failed to provide conclusive evidence in support of this hypothesis. Further research is therefore warranted to better disentangle under which conditions intranasal oxytocin has a reliable effect.

### Limitations

One limitation of our study is that we were not able to test oxytocin effects on mimicry of anger. Given that an affiliation intent is necessary for emotional mimicry (Fischer and Hess, 2017), and that angry expressions may be interpreted as a threat signal, our task may not have provided a suitable context for mimicry of anger to occur. To test whether oxytocin effects on mimicry are emotion-specific, future studies should present additional emotional expressions, and measure facial reactions in more interactive paradigms that promote affiliative goals.

Even though the sample size of this study was similar to other oxytocin studies (e.g., Korb et al., 2016), it had insufficient power to detect small-to-medium effects, which are the effect sizes typically observed in oxytocin research (Walum et al., 2016). As evidenced by the inconclusive results from the equivalence tests, further studies with higher statistical power are needed to draw reliable conclusions about the presence or absence of a meaningful effect of oxytocin on facial mimicry. Prospective research would benefit from determining a-priori the smallest effect size of interest for equivalence testing. Even though setting the equivalence boundaries according to the sample size of this study helped us to determine that our data was not sensitive enough to detect even large effects, a better approach would derive more precise and meaningful theoretical predictions based on prior studies, instead of on a resource question (Lakens et al., 2018). Also, the inclusion of female participants in future studies is encouraged, as so far all intranasal oxytocin studies on mimicry have exclusively tested men, and gender differences are commonly observed in oxytocin research (e.g., Lynn et al., 2014; Rilling et al., 2014).

Finally, while intranasal oxytocin is the most accessible and widespread method to study the role of oxytocin on human behavior, this methodology is not without limitations. First, even though oxytocin is administered intranasally to take advantage of the direct pathways between the nasal cavity and the central nervous system (Quintana et al., 2015), it is still unclear what doses are needed to reach relevant brain regions and induce behavioral effects (Leng and Ludwig, 2016). Compared to 12 IU and 40 IU, the dose administered here (24 IU) exerted the maximum impact on neural reactivity in a study on dose-dependency (Spengler et al., 2017). However, dose-dependent effects were not observed on the behavioral level. Moreover, it is yet to be determined to what extend oxytocin’s central actions account for its socio-cognitive effects, or whether the peripheral oxytocinergic system is also involved (Quintana et al., 2015; Leng and Ludwig, 2016; Valstad et al., 2016). Further research is thus warranted to improve our understanding of the oxytocinergic system and to develop improved methods to study the role of oxytocin on social behavior and cognition.

### Conclusion

Results from this study add to the evidence that facial mimicry is influenced by the reward value of the interactant, and reinforces the notion of mimicry as a context-specific social process. Nevertheless, the fact that the reward effects were in the opposite direction as reported in previous mimicry studies highlights the need to closely evaluate the impact of experimental protocols, sample characteristics, and contextual factors on the modulation of mimicry by reward. As shown by other replication attempts in psychological science, small differences in study designs may lead to meaningful changes in the results (Noah et al., 2018). Rather than invalidating the reported findings, failed replications should be taken as an opportunity to identify new possible moderators of the investigated effects (Van Bavel et al., 2016).

Also, we did not find evidence for a significant role of oxytocin on the effect of reward on mimicry in response to happy expressions. While this is the first time, to our knowledge, that the influence of oxytocin on the reward-mimicry link is investigated, our results coincide with previous reports of null effects of oxytocin on mimicry of happiness. Nevertheless, the sample size of this study, which limited the power to detect small or medium effects, and the fact that only male participants were included, warrant a cautious interpretation and generalizability of these null results. As in other fields, oxytocin research has suffered from strong publication bias (Lane et al., 2016), and some of the early findings have not been replicated (e.g., Nave et al., 2015). Parallel to efforts in improving the methodological quality of oxytocin studies (Walum et al., 2016), it is warranted that failed replications and null results like this one are brought to light and taken into account when assessing the role of oxytocin on social cognitive processes.

## Data Availability Statement

All datasets generated for this study are available at: https://osf.io/n85sh/.

## Ethics Statement

The studies involving human participants were reviewed and approved by Ethics Committee of the department of Psychology at Humboldt-Universität zu Berlin. The patients/participants provided their written informed consent to participate in this study. Written informed consent was obtained from the individuals for the publication of any potentially identifiable images or data included in this article.

## Author Contributions

All authors contributed to the conception of the study. IT, HD, and ID designed the study. IT collected the data, performed the statistical analyses, and wrote the first draft of the manuscript. All authors contributed to manuscript revision, read and approved the submitted version.

## Conflict of Interest

The authors declare that the research was conducted in the absence of any commercial or financial relationships that could be construed as a potential conflict of interest.
