# Supplementary material for: The Influence of Reward on Facial Mimicry: No Evidence for a Significant Effect of Oxytocin
Source: Front Behav Neurosci. 2020 Jun 12;14:88. doi: 10.3389/fnbeh.2020.00088 (PMC7304412; doi:10.3389/fnbeh.2020.00088)
Supplement: Supplementary file 1 [file Table_1.DOCX]

Supplementary Material

**Table S1**. Mean (standard deviation) of questionnaire scores for the full sample, and for each treatment group.

| Measurements |  | Full sample  (n = 60) | Placebo  (n = 30) | Oxytocin  (n = 30) |
| --- | --- | --- | --- | --- |
| Autism Spectrum Quotient |  | 8.40 (4.69) | 8.87 (4.01) | 7.93 (5.31) |
| Empathy Quotient |  | 36.33 (10.49) | 36.10 (11.08) | 36.57 (10.04) |
| Mehrfachwahl-Wortschatz-Intelligenztest-B | | 26.67 (4.58) | 27.33 (4.82) | 26.00 (4.32) |
| Positive affect scale (PANAS) | Pre-treatment administration | 30.98 (6.91) | 30.27 (7.70) | 31.70 (6.08) |
|  | Post-treatment administration | 30.47 (6.95) | 29.97 (7.49) | 30.97 (6.46) |
| Negative affect scale (PANAS) | Pre-treatment administration | 14.02 (4.62) | 14.13 (5.08) | 13.90 (4.20) |
|  | Post-treatment administration | 12.60 (3.97) | 12.73 (4.77) | 12.47 (3.05) |

*Note*: PANAS: Positive and Negative Affect Schedule.

**Table S2**. Mean (standard deviation) ﻿﻿proportion of high reward choices in each of the first three trial blocks of the reward learning task.

|  | Full sample  (n = 60) | Placebo  (n = 30) | Oxytocin  (n = 30) |
| --- | --- | --- | --- |
| Block 1 | ﻿0.60 (0.19) | ﻿0.59 (0.19) | ﻿0.60 (0.19) |
| Block 2 | ﻿0.72 (0.21) | ﻿0.70 (0.21) | ﻿0.73 (0.22) |
| Block 3 | ﻿0.75 (0.20) | ﻿0.73 (0.21) | ﻿0.77 (0.19) |

**Table S3**. Mean pleasantness ratings (standard deviation) for the faces paired with low and high reward values, as rated before and after the reward learning task.

|  |  | Full sample  (n = 60) | Placebo  (n = 30) | Oxytocin  (n = 30) |
| --- | --- | --- | --- | --- |
| Pre-reward learning | Low reward | 3.72 (0.71) | ﻿3.75 (0.76) | ﻿3.68 (0.66) |
|  | High reward | 3.77 (0.89) | ﻿3.85 (0.78) | ﻿3.68 (0.99) |
| Post-reward learning | Low reward | 3.76 (0.91) | ﻿3.80 (0.95) | ﻿3.72 (0.88) |
|  | High reward | 4.23 (0.98) | ﻿4.42 (0.81) | ﻿4.05 (1.12) |

**Table S4**. Mean (standard deviation) EMG scores for each recorded muscle, as a function of treatment group, reward and emotion conditions. EMG scores refer to Z-standardized, baseline-corrected EMG data for the interval between 1500 and 4000 ms post-stimulus onset.

|  |  | Placebo (n = 27) | | Oxytocin (n = 29) | |
| --- | --- | --- | --- | --- | --- |
|  |  | Zygomaticus major | Corrugator supercilii | Zygomaticus major | Corrugator supercilii |
| Happy expressions | No reward | 0.02 (1.08) | -0.03 (0.86) | 0.08 (1.10) | -0.08 (0.93) |
|  | Low reward | 0.11 (0.97) | -0.04 (0.76) | 0.12 (0.73) | -0.08 (0.71) |
|  | High reward | 0.03 (0.85) | -0.06 (0.74) | 0.02 (0.85) | -0.03 (0.70) |
| Angry expressions | No reward | 0.04 (1.38) | -0.02 (0.88) | 0.005 (1.75) | 0.01 (0.78) |
|  | Low reward | 0.04 (0.85) | 0.0001 (0.62) | 0.05 (0.75) | -0.02 (0.81) |
|  | High reward | -0.04 (0.76) | -0.01 (0.87) | 0.03 (0.96) | -0.06 (0.76) |

**Table S5**. Results of the linear mixed models conducted to test the effect of treatment (placebo vs. oxytocin) on EMG scores for each recorded muscle (zygomaticus major vs. corrugator supercilii) in response to expressions of happiness (Model 1) and anger (Model 2) by unconditioned faces (i.e. faces not presented in the reward learning task).

|  | **Model 1: EMG responses to happy expressions** | | | | | |  | **Model 2: EMG responses to angry expressions** | | | | | |
| --- | --- | --- | --- | --- | --- | --- | --- | --- | --- | --- | --- | --- | --- |
|  | *Estimate* | *SE* | *95% CI* | *ß* | *t* | *p* |  | *Estimate* | *SE* | *95% CI* | *ß* | *t* | *p* |
| Muscle | 0.05 | 0.02 | 0.02 – 0.09 | 0.07 | 3.04 | **.002** |  | 0.01 | 0.02 | -0.03 – 0.06 | 0.02 | 0.64 | .522 |
| Treatment | 0.06 | 0.02 | 0.02 – 0.10 | 0.08 | 2.71 | **.007** |  | -0.003 | 0.03 | -0.05 – 0.05 | -0.003 | -0.11 | .910 |
| Muscle x Treatment | -0.03 | 0.02 | -0.06 – 0.01 | -0.04 | -1.65 | .100 |  | 0.02 | 0.02 | -0.03 – 0.06 | 0.02 | 0.82 | .414 |
| **Random Effects** |  | | | | | |  |  | | | | | |
| σ^2^ | 0.51 | | | | | |  | 0.82 | | | | | |
| τ_00_ | 0.01 _participant,_ 0.003 _stimuli_ | | | | | |  | 0.01 _participant_ | | | | | |
| ICC | 0.02 | | | | | |  | 0.01 | | | | | |
| N | 56 _participant,_ 6 _stimuli_ | | | | | |  | 56 _participant_ | | | | | |
| Observations | 1734 | | | | | |  | 1706 | | | | | |
| Marginal R^2^/ Conditional R^2^ | 0.013 / 0.035 | | | | | |  | 0.001 / 0.013 | | | | | |

*Note*: *p*-values for the fixed effects calculated using Wald-statistics approximation, uncorrected. Significant *p*-values according to alpha < 0.05 are indicated in bold. Model equation: EMG scores ~ Muscle + Treatment + Muscle*Treatment + (1|Participant) + (1|Stimuli). EMG scores refers to the Z-standardized, baseline-corrected EMG activity for each muscle, averaged within the interval between 1500 and 4000 ms post-stimulus onset. The inclusion of the random intercept for stimuli in Model 2 lead to singular fits and was therefore removed from the final model. Estimate: unstandardized coefficient; SE: standard error; CI: confidence interval; *ß*: standardized coefficient; *t*: test statistic coefficient; *p*: p-value; σ^2^: within-group variance; τ_00_ = between-group variance; ICC = interclass correlation (ratio of between-cluster variance to total variance); N: number of random effects.

**Table S6**. Results of the linear mixed models conducted to test the effect of treatment (placebo vs. oxytocin) on EMG scores for each recorded muscle (zygomaticus major vs. corrugator supercilii) in response to expressions of happiness (Model 3) and anger (Model 4) by faces previously associated with low reward and high reward.

|  | **Model 3: EMG responses to happy expressions** | | | | | |  | **Model 4:EMG responses to angry expressions** | | | | | |
| --- | --- | --- | --- | --- | --- | --- | --- | --- | --- | --- | --- | --- | --- |
|  | *Estimate* | *SE* | *95% CI* | *ß* | *t* | *p* |  | *Estimate* | *SE* | *95% CI* | *ß* | *t* | *p* |
| Muscle | 0.06 | 0.01 | 0.03 – 0.08 | 0.08 | 4.37 | **<.001** |  | 0.02 | 0.01 | -0.01 – 0.05 | 0.03 | 1.48 | .140 |
| Reward | 0.02 | 0.01 | -0.01 – 0.05 | 0.03 | 1.50 | .134 |  | 0.02 | 0.01 | -0.01 – 0.04 | 0.02 | 1.26 | .209 |
| Treatment | 0.03 | 0.02 | -0.02 – 0.07 | 0.04 | 1.24 | .215 |  | -0.004 | 0.02 | -0.03 – 0.03 | -0.01 | -0.28 | .777 |
| Muscle x Reward | 0.03 | 0.01 | 0.00 – 0.05 | 0.04 | 2.10 | **.036** |  | 0.01 | 0.01 | -0.02 – 0.03 | 0.01 | 0.53 | .594 |
| Muscle x Treatment | -0.002 | 0.01 | -0.03 – 0.02 | -0.002 | -0.12 | .902 |  | -0.02 | 0.01 | -0.05 – 0.01 | -0.03 | -1.43 | .152 |
| Reward x Treatment | 0.005 | 0.01 | -0.02 – 0.03 | 0.01 | 0.36 | .719 |  | 0.004 | 0.01 | -0.02 – 0.03 | 0.01 | 0.28 | .777 |
| Muscle x Reward x Treatment | -0.01 | 0.01 | -0.04 – 0.01 | -0.02 | -0.85 | .397 |  | 0.01 | 0.01 | -0.02 – 0.04 | 0.02 | 0.85 | .397 |
| **Random Effects** |  | | | | | |  |  | | | | | |
| σ^2^ | 0.53 | | | | | |  | 0.53 | | | | | |
| τ_00_ | 0.01 _participant_, 0.0004 _stimuli_ | | | | | |  | 0.003 _participant,_ 0.0003 _stimuli_ | | | | | |
| ICC | 0.03 | | | | | |  | 0.01 | | | | | |
| N | 51 _participant,_ 6 _stimuli_ | | | | | |  | 51 _participant,_ 6 _stimuli_ | | | | | |
| Observations | 2966 | | | | | |  | 2944 | | | | | |
| Marginal R^2^/ Conditional R^2^ | 0.010 / 0.035 | | | | | |  | 0.002 / 0.008 | | | | | |

*Note*: *p*-values for the fixed effects calculated using Wald-statistics approximation, uncorrected. Significant *p*-values according to alpha < 0.05 are indicated in bold. Model equation: EMG scores ~ Muscle + Reward + Treatment + Muscle*Reward + Muscle*Treatment + Reward*Treatment + Muscle*Reward*Treatment + (1|Participant) + (1|Stimuli). EMG scores refers to the Z-standardized, baseline-corrected EMG activity for each muscle, averaged within the interval between 1500 and 4000 ms post-stimulus onset. Estimate: unstandardized coefficient; SE: standard error; CI: confidence interval; *ß*: standardized coefficient; *t*: test statistic coefficient; *p*: p-value; σ^2^: within-group variance; τ_00_ = between-group variance; ICC = interclass correlation (ratio of between-cluster variance to total variance); N: number of random effects.
